# Chocolate unicorns and smiling teddy biscuits: analysis of the use of child-directed marketing on the packages of Australian foods

**DOI:** 10.1017/S136898002300215X

**Published:** 2023-12

**Authors:** Alexandra Jones, Maria Shahid, Georgia Morelli, Kylie Howes, Devorah Riesenberg, Katherine Sievert, Simone Pettigrew, Gary Sacks

**Affiliations:** 1 The George Institute for Global Health, University of New South Wales, UNSW, Sydney, Australia; 2 Global Centre for Preventive Health and Nutrition, Institute for Health Transformation, Faculty of Health, Deakin University, Geelong, Australia

**Keywords:** Marketing, Food packaging, Child, Nutritional quality

## Abstract

**Objective:**

The use of food packaging as a vehicle for marketing to children is under investigated. Our objective was to determine the prevalence and types of child-directed promotional techniques used on food packaging in Australia.

**Design:**

Based on existing literature and regulations, we developed a framework to classify on-pack child-directed promotional techniques involving the use of characters and other elements that appeal to children. We analysed the packaging of all products in eight food categories available for sale from supermarkets in 2019 and recorded the use of child-directed promotions on pack. We assessed the number and proportion of products displaying child-directed promotional techniques overall and assessed the healthiness of products using child-directed promotions against four indicators of healthiness to provide summary data overall and for the manufacturers who most frequently employed child-targeted strategies.

**Setting:**

Data were collected from the FoodSwitch database in Sydney, Australia.

**Results:**

901/8006 (11·3 %) products displayed one or more child-directed on-pack element. Most frequent was on foods for infants and young children (*n* 315), confectionery (*n* 283), snack foods (*n* 172) and dairy (*n* 168). Personified characters were the most commonly used element (*n* 512). Products using child-directed promotional techniques scored poorly on all four indicators of healthiness: mean health star rating 2·34 (out of 5); 81 % ultra-processed and 6·1 % and 4·5 % products eligible to market to children under Western Pacific and Mexican nutrient profiling schemes, respectively.

**Conclusions:**

Australian children are targeted by promotional techniques on the packaging of unhealthy food products. Stronger regulation of these techniques is warranted to protect children’s health.

Too many Australian children are consuming unhealthy diets that are high in added sugars, saturated fats, salt, energy and ultra-processed foods, with inadequate intake of fruits, vegetables and wholegrains^(1)^. Unhealthy diets have lifelong adverse health consequences. Childhood overweight and obesity is a major public health issue in Australia, affecting approximately one in four children and adolescents^(2)^. Living with overweight or obesity as a child is associated with increased risk of developing conditions like dental caries, type two diabetes, heart disease and some cancers, as well as remaining overweight as an adult, which further adds to the risk of disease^(3)^. Children living with overweight and obesity may also experience psychological and psychosocial impacts such as weight stigma and low self-esteem^(4)^.

Unprecedented availability and aggressive marketing of ultra-processed, packaged foods and beverages is a key driver of childhood obesity and diet-related conditions^(5)^. There is strong empirical and review evidence that children’s exposure to food marketing leads to cascading effects, including on brand awareness, positive brand attitudes and purchase and consumption behaviours^(6)^. Consumer behaviour patterns begin very early in life and develop in tandem with other aspects of motor and cognitive development. For example, there is evidence that children as young as 18 months can recognise corporate labels, at 20 months can associate items with brand names, at 2 years old can make consumer choices and by 2–3 years can draw brands^(7)^. The centrality of children to household decision making and their vulnerability to food promotion leaves parents (usually mothers) as adjudicators between competing desires and demands^(8)^. The influence of marketing can be seen, for example, in the recognised concept of ‘pester power’, by which children can be encouraged to demand marketed products and influence purchasing decisions of their caregivers^(9,10)^. Among its suite of evidence-based policies to promote healthier diets, the WHO recommends implementing marketing restrictions on unhealthy foods and beverages^(11,12)^. In Australia, to date, restrictions on food marketing have largely been set and regulated through self-regulatory codes developed by the food and advertising industries that apply to specific types of media (i.e. television, print, radio, cinema, outdoor advertising and internet sites)^(13)^. The only government regulation of advertising to children applies exclusively to free-to-air television and has limited provisions related to food marketing specifically^(14)^. There is substantial evidence that this regulatory mix is inadequate to protect children’s health, and that stronger legislative action is needed^(15,16)^.

One area of increased regulatory interest internationally is the use of innovative restrictions on promotional techniques that appeal to children on the packaging of unhealthy foods. Such restrictions recognise that food packaging provides a critical opportunity for influencing consumers at the point of purchase and during consumption^(17)^. There is evidence that the use of techniques such as cartoon and movie characters, gifts, games and contests on product packages encourages children to think of these products as tasty, more fun and more appropriate for them, whilst increasing the likelihood of their selection and consumption^(18–21)^. Previous audits of the use of cartoon characters on food packaging, including Australian work from 2006 and 2011, suggest that these promotional techniques are more commonly employed on ‘unhealthy’ foods^(22,23)^. The influence of cartoon characters on children’s perceptions appears stronger among younger children (i.e. is higher for 6–8-year-olds compared with 9–12-year-olds) given their reduced cognitive ability to differentiate these characters from reality^(18,24)^.

To address the harms of children’s exposure to these specific promotional techniques, countries such as Peru, Chile and, most recently, Mexico have implemented legislation that seeks to limit the use of a range of child-directed promotional techniques on product packaging^(25–27)^. These requirements are part of broader regulatory packages that require unhealthy products (as defined by cut-off thresholds for risk nutrients such as salt, added sugars and saturated fats) to display front-of-pack ‘stop sign’ style warning labels. Products displaying warning labels are consequently not allowed to be marketed to children through a variety of media channels, including platforms conventionally targeted by marketing restrictions such as television and billboards, but also innovatively including the product’s packaging. These policies have been heralded as ‘world leading’ by public health groups and are starting to demonstrate encouraging results^(28–31)^, but have simultaneously provoked threatened and actual litigation from some food industry stakeholders. Although to date none of these challenges have been successful^(32,33)^, they have potential to create ‘regulatory chill’, whereby governments are dissuaded from progressive public health policymaking^(34)^.

In light of these regulatory developments and concerns around the exposure of children to unhealthy food marketing, the aim of this paper was to explore the frequency and types of child-directed promotional techniques used on food packaging in Australia in selected product categories. The goal was to provide an updated systematic analysis of the use of these techniques in Australia, using a broad definition of child-directed promotion, to inform potential regulation in Australia. The results are likely to be informative for regulators in other countries with similar food market characteristics.

## Methods

### Data source

The George Institute for Global Health’s FoodSwitch data collection and analysis platform captures images of food and beverage packaging using a bespoke mobile application, allowing for the extraction and collation of key food labelling and food composition data^(35)^. Using this process, the FoodSwitch Monitoring Datasets are generated annually based on systematic data collection from four large Australian supermarkets owned by Aldi, Coles, Independent Grocers of Australia and Woolworths in the Sydney metropolitan area. In-store data collection is undertaken by trained data personnel who photograph all food and beverage products to capture images of key information including barcodes, product name, front-of-pack nutrition labelling, health and nutrient content claims, package size, ingredients list, manufacturer and band names and the nutrition information panel. Data are entered into the Monitored Database using these images by trained data entry personnel. We used the Monitoring Dataset for 2019 for the purposes of this analysis due to delays in later collections resulting from COVID-related disruptions to store access.

### Product categorisation and selection of product categories

Categorisation of products in FoodSwitch is based on the system developed by the Global Food Monitoring Group^(36)^. A hierarchical category tree allows comparisons among nutritionally similar foods; products are categorised into major categories (e.g. bread and bakery products), minor categories (e.g. biscuits, breads) and further levels of sub-categories.

We used a targeted sample of product categories for this analysis, focusing on categories where child-directed marketing was more likely to be present. This involved identifying products with cartoon characters present, and then conducting a broader search into the categories from which those products came in FoodSwitch. We selected for further investigation the major categories where this tag appeared on more than ten products. Within these major categories, we excluded minor categories where a reasonable assumption could be made that child-directed marketing would not be used (i.e. excluding tea and coffee products from non-alcoholic beverages and excluding plain rices, grains and flours to leave a remaining focus on breakfast cereals in the cereal and grains category). Ultimately, this yielded eight focus categories for further analysis: biscuits, cakes, muffins and pastries, confectionery, breakfast cereals, non-alcoholic beverages, dairy, snack foods (e.g. potato chips, popcorn and muesli bars) and foods for infants and young children (these include baby foods and snacks specifically labelled as for children aged 0–36 months that are typically found in a baby food aisle).

Products were identified by their unique barcode. Where the same product (i.e. same item in same product size) appeared in more than one store surveyed, we counted it only once. Where a product appeared in more than one package size (i.e. had a different barcode), each package size was counted as an individual product, given the potential for different marketing techniques to be present on different package sizes.

### Use of child-directed promotional techniques

Existing literature and regulation were used to develop a framework of child-directed promotional techniques (see online Supplemental Table 1)^(30,37–41)^. Techniques were grouped into two major categories: ‘child-directed characters’ and ‘non-character-based elements that appeal to children’. Within these, ten specific promotional techniques were identified. Child-directed characters were grouped into those cartoons and/or fantastical characters that were licensed or branded, children or child-like figures, personified characters (e.g. spoons with faces) or celebrities that appeal to children. In identifying products that utilised specific types of ‘child-directed characters’, we also identified products that used characters that we determined were *not* child-directed, for example the adult male figure incorporated into the Quaker Oats logo. These ‘non-child-directed characters’ were not included in our analysis.

Non-character-based elements included childhood life references (e.g. playgrounds), gifts, games and contests that appeal to children, unconventional packaging or a product name specifically referencing children (e.g. ‘kids bar’). Some marketing elements required additional consideration when coding. For example, in assessing whether packaging included ‘children or child-like figures’, we used a threshold of whether the figure appeared <14 years old consistent with the age thresholds applied in recent Chilean and Mexican legislation. In assessing whether a celebrity or contest ‘appealed to children’, we coded additional detail on the name or nature of the celebrity or contest and two coders discussed additional contextual factors such as whether the celebrity was from a television program or movie watched by children, or whether the prize in a contest was a toy. In determining ‘appeal to children’, it was not necessary for the marketing to appeal *only* to children (e.g. a celebrity such as an Olympic swimmer could appeal to both adults and children) to meet our criteria. Each product could be coded for multiple marketing techniques.

Two coders examined photographs of product packaging in FoodSwitch and independently recorded the appearance of these techniques anywhere on the product package. Coding reliability was checked by the two coders cross-coding a random 10 % sample of the other coder’s completed categories. Percent agreement was >90 % for all product categories. Disagreements in coding, for example on whether a celebrity ‘appealed to children’, were resolved through discussion with both coders and a third coder.

### Assessment of product healthiness

We used nutritional information and pre-calculated indicators of healthiness contained in FoodSwitch to assess the healthiness of products in our sample using four different indicators of nutritional quality:

#### The Australasian Health Star Rating

The Health Star Rating (HSR) is a government-led front-of-pack nutrition labelling system, implemented on a voluntary basis in Australia since 2014^(42)^. It uses an algorithm to assign products an overall score from 0·5 to 5·0 stars in ten half-star increments. Where a product was displaying a HSR on its label, we used the HSR displayed by manufacturer for the purposes of our analysis. In cases where a HSR was not displayed, it was calculated in alignment with the methods provided by government guidance documents^(43)^. This involved (i) categorising the foods into one of six HSR categories (i.e. non-dairy beverages; dairy beverages; oils and spreads; cheese and processed cheese; all other dairy foods; all other non-dairy foods) and excluding those products government guidance deems are not ‘HSR eligible’ (for example, vitamins and minerals, alcohol and notably in this case foods for infants and young babies); (ii) calculating baseline points based on the energy, saturated fat, total sugar and sodium content per 100 g and (iii) modifying points for fruit, vegetable, nut and legume content and protein and fibre (where applicable) were calculated. Where details such as fruit, vegetable, nut and legume content were not provided by the manufacturer on the package, levels were estimated using information drawn from the back-of-pack ingredients list and generic food composition databases, or by analogy with similar products using methods described previously^(35)^. A HSR score was calculated by subtracting the modifying points from baseline points and converting this to a HSR as specified by policy guidance. For this project, we used pre-calculated HSR available in the FoodSwitch database.

#### NOVA classification of degree of processing

The NOVA classification system groups food products according to how much processing they have been through. The four groups are unprocessed and minimally processed foods (Group 1); culinary ingredients (Group 2); processed foods (Group 3) and ultra-processed foods (Group 4). NOVA is generally applied in the FoodSwitch database at the most granular level of sub-category, facilitating classification of nutritionally similar products. Where differences in NOVA classification were known to be present at a sub-category level (e.g. for packaged bread loaves, canned fruit or vegetable products), NOVA classification was assigned at an individual product level by searching the ingredients list to determine the presence or absence of ingredients found exclusively in ultra-processed foods (e.g. food additives, colours). For this project, we used pre-assigned NOVA classifications available in the FoodSwitch database.

#### WHO nutrient profiling model for the Western Pacific Region (WHO WPRO)

The WHO WPRO nutrient profiling model assessed whether a product is eligible or ineligible to be marketed to children. It takes into consideration total fat, total sugar, added sugar, non-sugar sweetener, energy, saturated fat and Na to determine eligibility of products across eighteen categories. For some categories (e.g. chocolates and sugar confectionery, cakes and biscuits, energy drinks, tea and coffee), all marketing to children is designated as prohibited, meaning no nutrient criteria are required. For this project, we used pre-calculated eligibility outcomes for the WHO WPRO criteria available in the FoodSwitch database.

#### Mexican nutrient profiling model for front-of-pack nutrition labelling and related marketing restrictions

We used the nutrient profile contained in Mexican legislation to determine whether products would be required to display one or more ‘stop sign’ warning labels and thereby also be restricted from using child-directed marketing techniques on pack^(27)^. We used information extracted from the nutrition information panel to quantify relevant nutrients plus searches of product ingredients lists to determine whether these amounts were from *added* sugars, fats and Na (as opposed to naturally occurring sources) to meet the terms of this legislation (see online Supplemental Table 2). Ingredients lists were also used to determine the presence of caffeine and non-nutritive sweeteners as required by the regulation. A list of non-nutritive sweeteners ingredients was drawn from previous work^(44)^. Application of the criteria from Mexican legislation FoodSwitch data was newly conducted for the purposes of this project. One analyst developed the code to run this analysis over the dataset, and this code was independently checked by a second FoodSwitch analyst to verify the reliability of results.

### Statistical analysis

We assessed the number and proportion of products displaying child-directed marketing technique(s) and the total number of child-directed marketing techniques used on pack for our overall sample and in each of the eight focus categories. To examine the nutritional quality of products with any form of child-directed marketing, we calculated the mean (95 % CI) HSR score; proportions of products across the four NOVA classification types and the proportions of products eligible to market to children under the WHO WPRO and Mexican nutrient profiling models. Lastly, we assessed the number of products and mean (95 % CI) HSR score of products with child-directed marketing made by the manufacturers with the highest number of products using child-directed marketing on pack.

Data manipulation and statistical analyses were conducted using Stata/BE 17.0 and figures generated using Microsoft Excel.

## Results

### Prevalence of child-directed marketing on product packaging

In total, 8006 products were surveyed across our eight focus categories. Of these, 901 products (11·3 %) displayed one or more child-directed promotional techniques on pack (Table 1).

**Table 1:** Number and proportion of products displaying one or more child-directed promotional technique on pack

	Number of products (*n*)	Proportion of total (%)
Products with no child-directed promotion	7105	88·7
Products with child-directed promotion	901	11·3
Total products in categories surveyed	8006	100·0

We coded 1156 instances of child-directed promotions. Among techniques used, child-directed characters were more than twice as common as non-character-based elements that appeal to children (*n* 794 and *n* 362 occurrences, respectively). The most prevalent techniques were personified characters (*n* 512), childhood life references (*n* 187), children and child-like figures (*n* 145), licensed or branded cartoon characters (*n* 124) and names that specifically reference children (*n* 93) (Table 2). Specific examples of child-directed marketing from the surveyed categories are shown in Table 3.

**Table 2:** Instances and types of child-directed promotional techniques being used overall and across each category surveyed

Food category	A1	A2	A3	A4	B1	B2	B3	B4	B5	B6	Total
Foods for infants and young children	0	110	119	0	28	0	3	0	0	55	**315**
Confectionery	62	5	123	0	60	1	3	0	25	4	**283**
Snack foods	19	14	74	0	42	0	0	4	5	14	**172**
Dairy	18	7	90	0	30	0	0	8	1	14	**168**
Non-alcoholic beverages	1	0	43	11	8	0	0	3	8	0	**74**
Biscuits	3	4	47	0	9	0	0	2	3	5	**73**
Breakfast cereals	17	5	5	2	0	0	9	6	1	1	**46**
Cakes, muffins and pastries	4	0	11	0	10	0	0	0	0	0	**25**
**Total number**	**124**	**145**	**512**	**13**	**187**	**1**	**15**	**23**	**43**	**93**	**1156**

Type A promotions include child-directed characters: A1 – Licensed or branded cartoon and fantastical characters; A2 – Children and child-like figures; A3 – Personified characters; A4 – Presence of celebrities. Type B promotions includes non-character-based elements that appeal to children: B1 – Childhood life references; B2 – Gifts; B3 – Games; B4 – Contests; B5 – Unconventional packaging; B6 – Name specifically references children.

**Table 3:** Examples of child-directed promotional techniques from each surveyed category

Code level 1	Code level 2	Example from results
A. Child-directed characters	A1 – Child Directed characters – cartoon and fantastical characters	Aeroplane Jelly Original Berry Blue Flavour 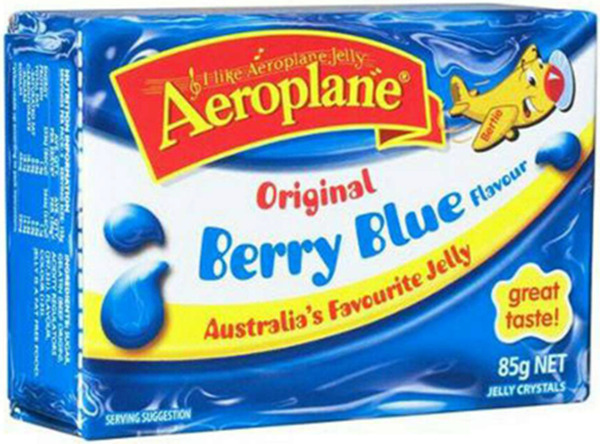
	A2 – Child Directed Characters – Children and child-like figures	Nestle Milo Duo 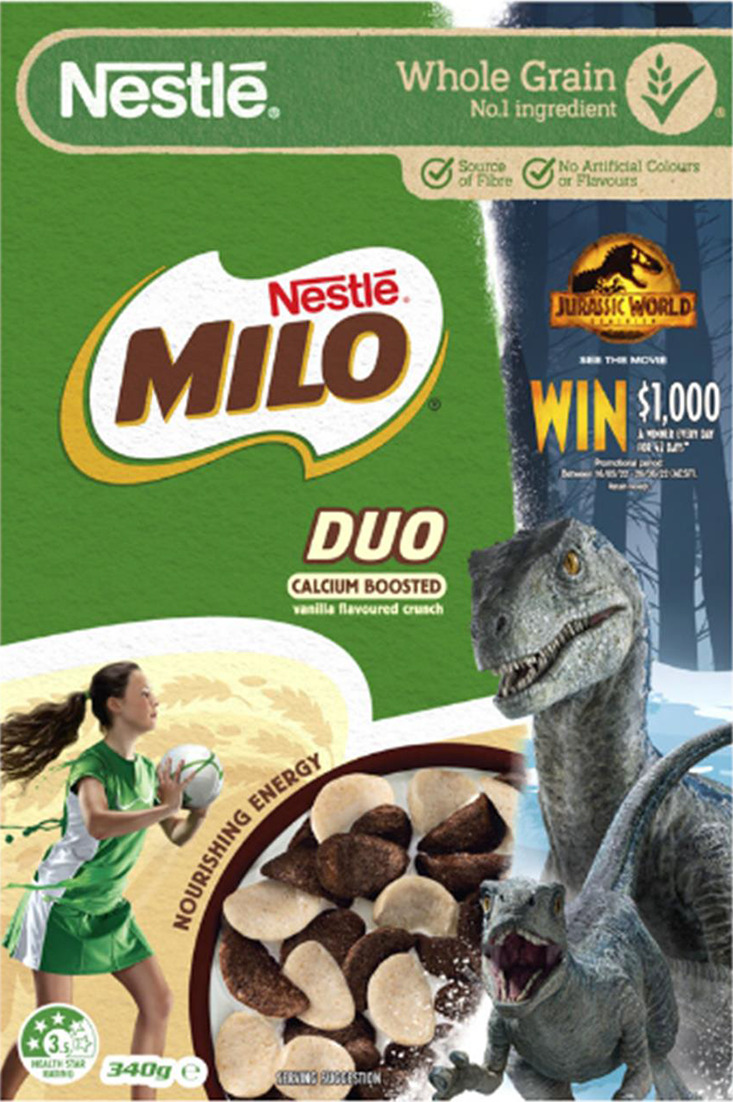
	A3 Child directed characters – Personified objects	Bega Stringers Tasty Cheddar Flavour 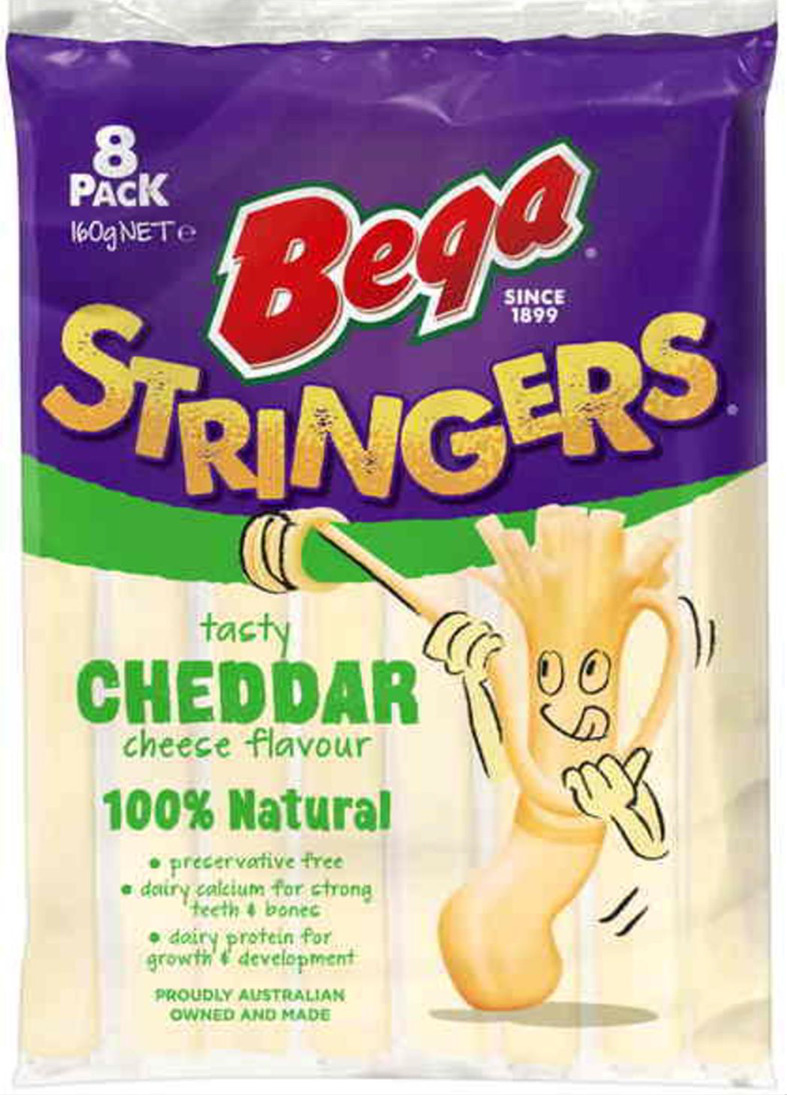
	A4 Child-directed marketing – Presence of celebrities	Nutri-grain pouches 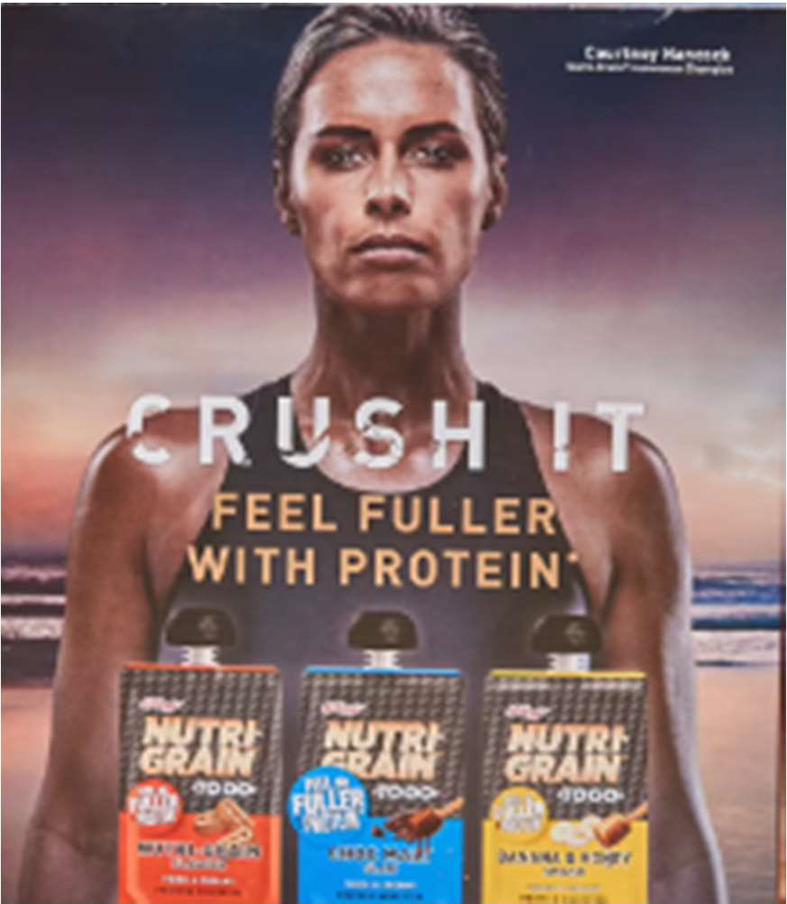
B. Non-character-based elements that appeal to children	B1 – Non-character-based elements that appeal to children – Childhood life references	Haribo Starmix 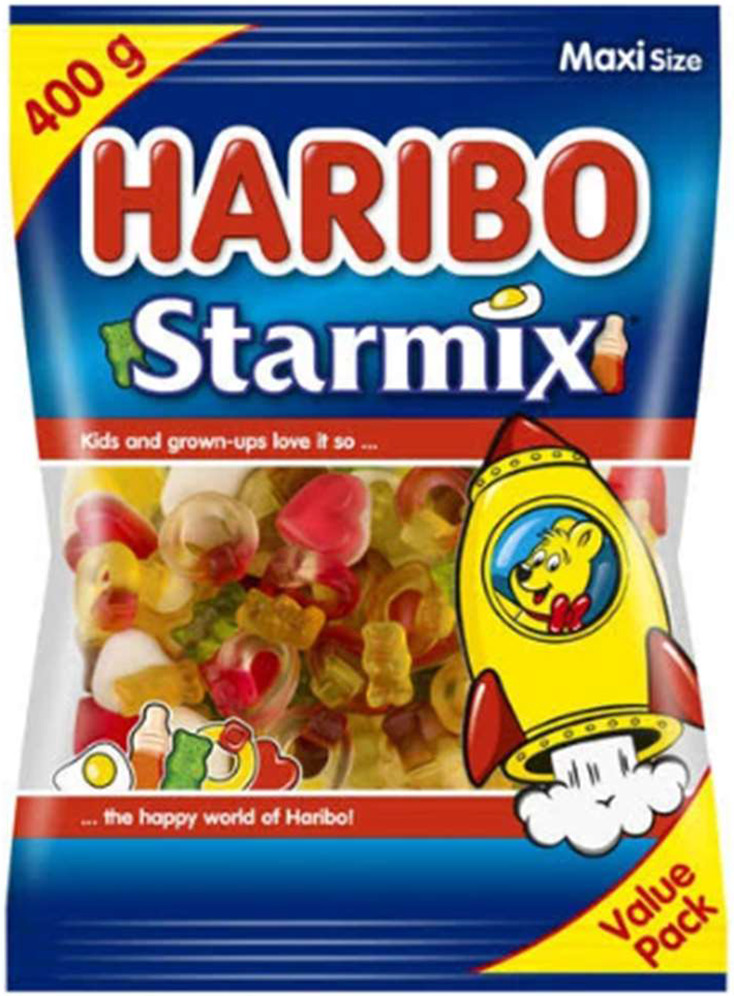
	B2 – Non-character-based elements that appeal to children – Gift	Kinder Surprise 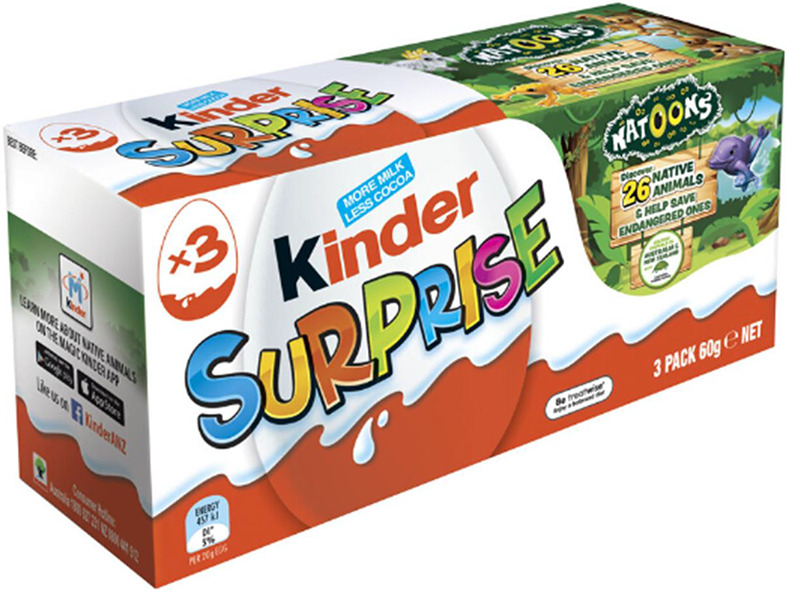
	B3 – Non-character-based elements that appeal to children – Games	Lowan Wholefoods Cocoa Bombs 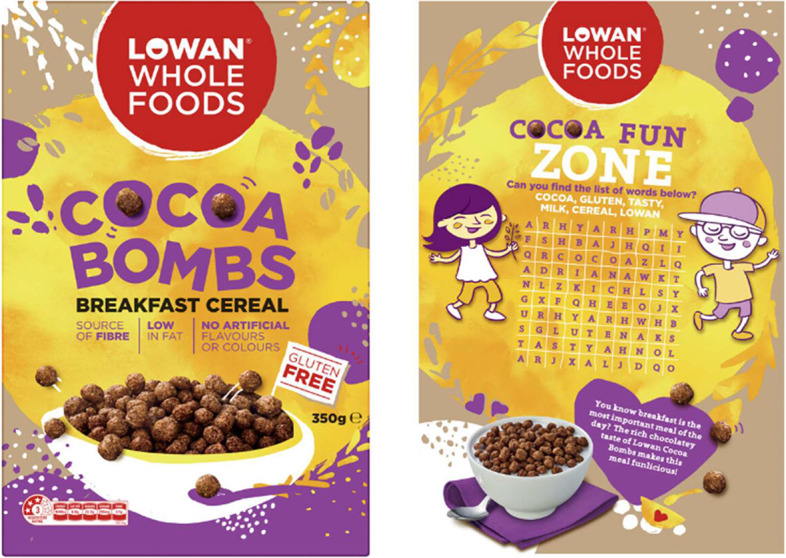
	B4 – Non-character-based elements that appeal to children – Contests	Doritos Corn Chips 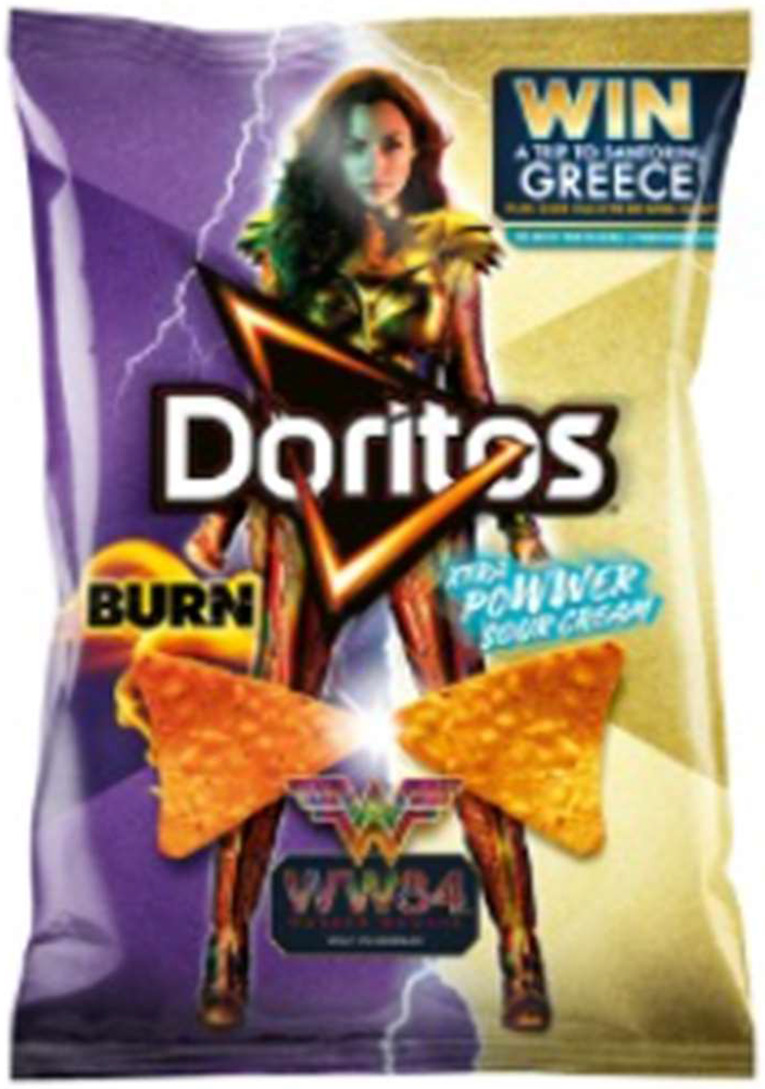
	B5 – Non-character-based elements that appeal to children – Unconventional packaging	Coles Musk Sticks 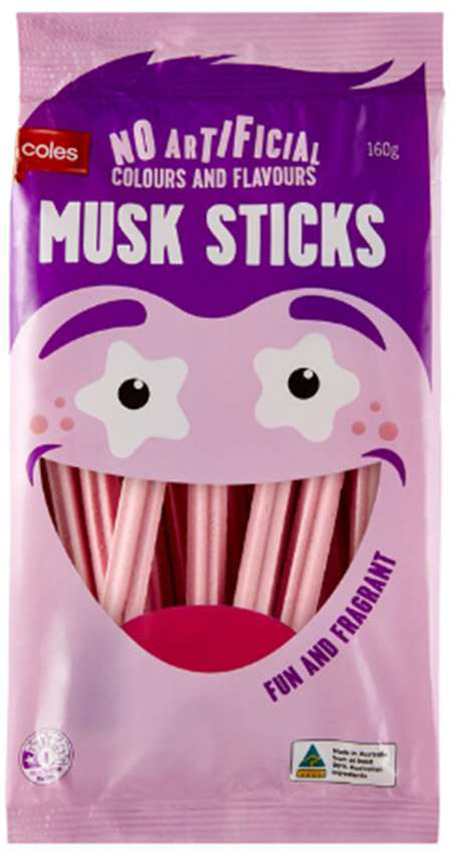
	B6 – Non-character-based elements that appeal to children – Name specifically references children	Vaalia Probiotics Kids Banana yoghurt 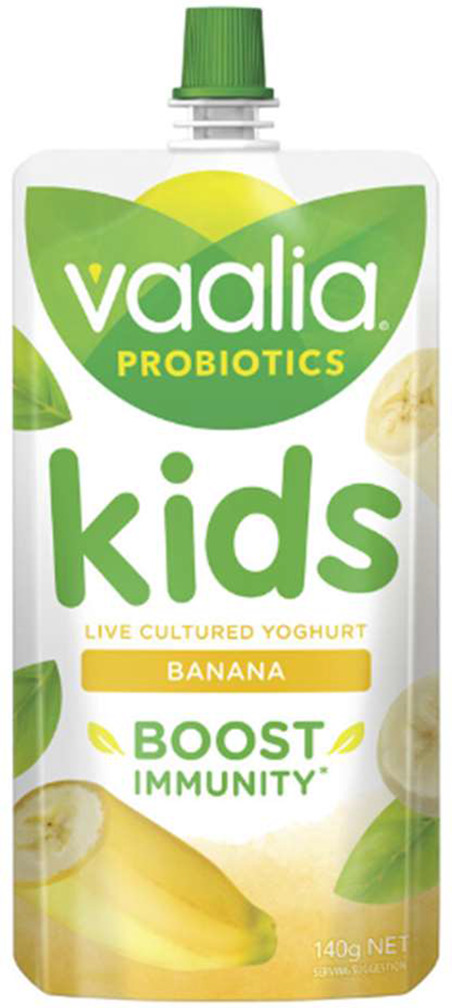

**Fig. 1:**
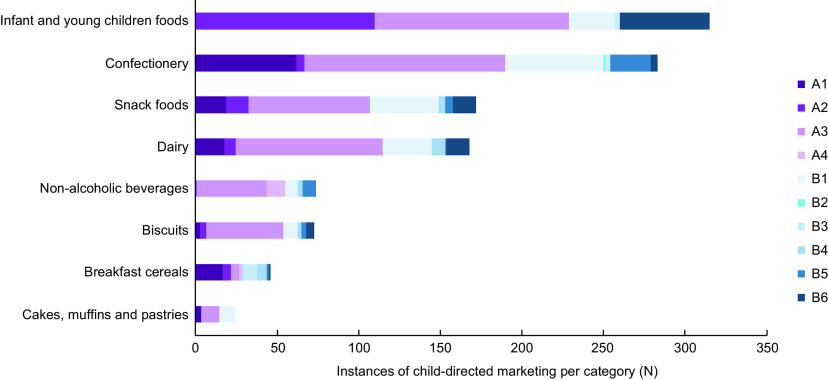
Instances and types of child-directed marketing techniques used across each category surveyed Type A products include child-directed characters: A1 – Licensed or branded cartoon and fantastical characters; A2 – Children and child-like figures; A3 – Personified characters; A4 – Presence of celebrities. Type B products include non-character-based elements that appeal to children: B1 – Childhood life references; B2 – Gifts; B3 – Games; B4 – Contests; B5 – Unconventional packaging; B6 – Name specifically references children.

Among the eight categories, child-directed promotional techniques were most prevalent on infant and young-child foods (*n* 315), confectionery (*n* 283), snack foods (*n* 172) and dairy products (*n* 168). Figure 1 illustrates the number and type of child-directed marketing techniques across each category surveyed. Variation can be seen within categories, for example foods for infants and young children foods were more likely to use images of children and child-like figures, whereas confectionery had greater use of licensed and branded characters.

### Healthiness of products using child-directed promotions on product packaging

**Fig. 2:**
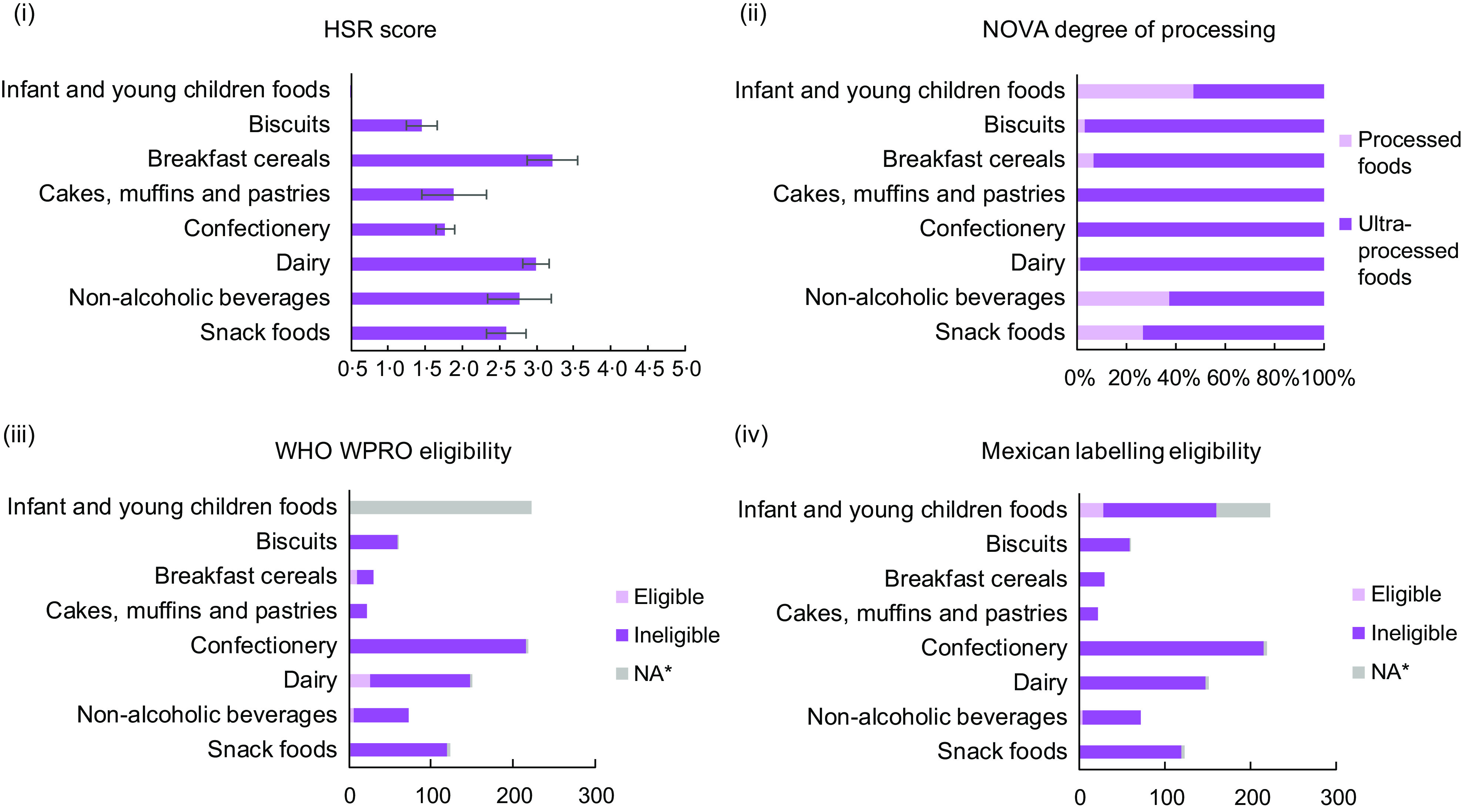
Nutritional quality of products with child-directed marketing as per four indicators of healthiness, by product category (i) mean Health Star Rating (HSR) score for HSR eligible products (n = 668), error bars represent the 95% confidence interval; (ii) proportion of products across each NOVA classification (Group 3: processed foods; Group 4: ultra-processed foods); (iii) number of products in/eligible for marketing to children under the World Health Organization (WHO)Western Pacific Region Office (WPRO) guidelines, *not applicable for assessment of eligibility; (iv) number of products in/eligible for marketing to children under the Mexican labelling legislation, *not applicable for assessment of eligibility.

The healthiness of products with and without child-directed promotional techniques on pack is presented in Fig. 2 for each of the four indicators of nutritional quality. Products using child-directed promotional techniques received poor scores on all four indicators. The mean HSR score of products with child-directed promotions on pack was 2·34, and the proportion of products NOVA-classified as ultra-processed with child-directed marketing on pack was 81·0 %. Few products were eligible to be marketed to children under either the WHO WPRO or Mexican labelling criteria. Among those products currently using child-directed promotions on pack, 6·1 % would be eligible to do so if applying the WHO WPRO model and 4·5 % would be adequately healthy to use these techniques if the Mexican criteria were applied (i.e. 95·5 % of products would be required to carry one or more warning labels under the Mexican legislation and would therefore be ineligible to market to children on pack or elsewhere).

Fifteen manufacturers produced nearly two-thirds (64·1 %) of all identified products with child-directed marketing on pack (Table 4). Among these, Aldi, Nestlé and Rafferty’s Garden had the largest proportions of products that utilised child-directed marketing on their packaging. Products with child-directed promotional techniques on pack made by Nudie Foods, Rafferty’s Garden and Parmalat had higher HSR scores (means 4·56, 3·83 and 3·40, respectively) than those made by manufacturers such as Mars and Kellogg’s (1·05 and 2·71, respectively).

**Table 4:** Total number of products, number and mean instances of child-directed promotional techniques per product and mean health star rating (HSR) score for the manufacturers using child-directed promotions most frequently

Manufacturer	Total products with child-directed promotions	Total instances of child-directed promotions across all products	HSR eligible* products with child-directed promotions		
	*n*	*n*	*n*	Mean HSR	95 % CI
Aldi†	76	90	58	2·42	2·12, 2·73
Nestlé	52	60	45	1·99	1·72, 2·26
Rafferty’s Garden	48	55	3	3·83	3·51, 4·16
Coles†	44	52	36	1·86	1·50, 2·22
Mondelēz	44	50	43	1·41	1·19, 1·62
Woolworths†	43	55	37	2·72	2·21, 3·22
Mars	38	45	38	1·05	0·81, 1·29
McCormick Foods	37	65	37	3·08	2·95, 3·22
Unilever	28	29	28	2·25	2·00, 2·50
Nudie Foods	25	27	25	4·56	4·09, 5·03
Bute Island Foods	23	23	0	–	
Vesco Foods	19	27	0	–	
Lion	17	17	17	3·71	3·32, 4·09
Parmalat	15	15	13	3·40	3·04, 3·76
Kellogg	14	20	14	2·71	2·29, 3·14
Campbell Arnott’s	13	13	11	1·50	1·20, 1·80
Metro Food Company	13	17	0	–	
Pringles	13	20	13	1·31	1·17, 1·45
The Kids Food Company	12	27	2	2·75	0·30, 5·20
Nourish Foods	11	22	6	2·75	1·62, 3·88
The Happy Snack Company	11	17	11	5·00	–
The Infant Food Company	11	31	0	–	
Heinz	10	15	0	–	
The Smith’s Snackfood Company	10	15	9	1·72	1·11, 2·34
All other manufacturers	274	349	220	2·20	2·02, 2·37

* As per government guidance on HSR, some categories of food are not eligible to carry a HSR.

† Denotes retailers. 95 % CI not listed (–) for means without variance (i.e. all values are the same).

Included manufacturers had at least ten products featuring any form of child-directed promotional techniques on pack.

These include vitamins and minerals, alcohol products and foods for infants and young children.

As a result, mean HSR results in this table exclude products made by these manufacturers in this category.

## Discussion

This study analysed the use of promotional techniques that appeal to children on the packaging of various categories of Australian supermarket products. We found that children are commonly targeted by promotions on the packages of unhealthy products in these categories.

Numerous promotional techniques were found to be used to increase the appeal of food to children. These included character-based elements such as personified objects (e.g. waving marshmallows with smiling faces), photographs or illustrations of children and licensed or branded cartoon characters (e.g. Minions, Bluey and the Coco Pops Monkey). Non-character-based techniques included childhood life references (e.g. references to ‘fun’, depiction of playground equipment), gifts (e.g. toys), games, contests, unconventional packaging (e.g. use of special shapes) and names that specifically reference children (e.g. ‘kids’ or ‘child’ in product name).

Instances of child-directed promotions on packages varied by category, but were particularly common in foods for infants and young children and confectionery categories in our analysis. The potential impact of child-directed promotions on infant and young child foods may be of particular ethical concern given the vulnerability of targeted consumers, and the fact that many of these products (e.g. extruded snacks for toddlers) are not a necessary part of a healthy diet for young children. The association of ‘fun’ or benign-looking characters may idealise these often unhealthy products, begin to influence very young children’s brand awareness and preferences and as they continue to develop can encourage ‘pester power’, thereby undermining optimal infant and young child feeding^(7,45)^. In the United Kingdom, the government’s Scientific Advisory Council on Nutrition has recently recommended that commercially manufactured foods and drinks marketed specifically for infants and young children are not needed to meet the nutritional requirements of this age group^(46)^. As part of broader concerns about the labelling and composition of foods in this category, United Kingdom authorities are currently considering the need for better packaging guidance to ensure clear, consistent and honest labelling and marketing of these products to address both deceptive marketing on nutritional quality and child-directed content^(47)^. There is some early indication that Australian authorities may do the same^(48)^.

Our results are broadly consistent with previous research showing that child-directed promotional techniques are predominantly used on less healthy foods^(22,23,39)^. Our work updates and expands Australian audits conducted in 2006^(23)^ and 2011^(39)^ to analyse the continued use of these promotional techniques across an ever changing food supply, with the results suggesting ongoing concern about these practices and their potential impact on children’s health is justified.

Our analysis also builds on this earlier work by using four contemporary metrics of nutritional quality, as well as adding to previous work by including criteria specifically developed to determine whether a product is sufficiently healthy to market to children (WHO WPRO) and to market to children on packaging specifically (Mexican legislation). Very few products currently using child-directed promotions on pack would be eligible to do so if these criteria were applied to regulate packaging in Australia. For example, if Australia were to adopt similar legislation to Mexico, >95 % of products currently using child-directed promotional techniques in these categories would have to display one or more warning labels on pack to highlight the presence of high levels of specific nutrients and also remove these techniques from packaging.

The exclusion of product packaging from existing weak restrictions on unhealthy marketing to children in Australia suggests that further regulatory action is warranted in this area. Drawing inspiration from Chile and Mexico, this regulation could be integrated with front-of-pack labelling policies such that foods that score below a determined threshold HSR score would not be eligible to use child-directed marketing on pack. In Chile, regulation has been shown to significantly reduce the use of child-directed marketing on breakfast cereal packages displaying warning labels and also to incentivise reformulation to retain eligibility to use these techniques on pack^(30)^. Mexican legislation has built on lessons from Chile to close ‘loopholes’ that potentially incentivise manufacturers to simply replace sugars with non-nutritive sweeteners by ensuring that products that contain non-nutritive sweeteners are also ineligible to market to children^(49)^. To operate effectively in Australia, similar legislation would require the HSR to be made mandatory and thereby displayed on all products. In its current voluntary form, the HSR is still on less than half of all products^(50)^ and missing from most unhealthy foods^(51)^, limiting its potential as a public health tool.

In the absence of regulation, it is notable that nearly two-thirds of products in the assessed categories that featured child-directed promotional techniques were made by fifteen manufacturers. This offers some potential for action targeting specific manufacturers to request they voluntarily stop using such techniques on pack. This may be particularly true for large grocery retailers who are also manufacturers (i.e. Aldi, Woolworths and Coles) given international examples of retailers who have voluntarily acted in other jurisdictions (e.g. Lidl in the UK^(52)^) and leadership of these retailers in Australia in voluntarily applying the HSR regardless of the scores received on their private label products^(51)^. However, given the significant financial disincentives for most manufacturers to voluntarily abandon established sources of marketing revenue, government-led regulatory solutions will be needed to drive meaningful and sustained change.

### Strengths and limitations

The current study has several strengths, including its large sample size and care taken in coding a variety of child-directed promotional techniques that have been more recently subject to regulatory attention in other countries. There are also some limitations. The product sample used may not be nationally representative, and there may be variation in the products children are exposed to in different parts of Australia. We focused on eight specific product categories only, and thus did not capture the full extent of child-directed promotions across the food supply (including in a range of healthier product categories). Nevertheless, the categories we selected included those in which child-directed promotions were most likely prevalent. Second, we acknowledge debate about the validity of different classification tools to accurately identify healthy and less healthy foods. Our use of four different tools (that use a variety of nutrient-based, category-based and level of processing criteria) strengthens our consistent finding that child-directed marketing is predominantly used on less healthy foods. Finally, we recognise that efforts to define ‘child-directed’ promotions are inherently subjective. We sought to minimise bias by using a detailed coding framework and multiple independent coders, but could not fully eliminate subjective judgement. Given the constantly evolving nature of marketing, future studies may also investigate additional strategies developed by marketers that we have not captured. As the WHO moves towards recommendations that countries implement policies that restrict not only ‘child-directed marketing’ but all marketing ‘to which children are exposed’^(10)^, future studies could also explore whether and how this definition could be applied to restrict an even greater range of techniques currently used on food packaging.

## Conclusions

Children in Australia are targeted by promotional techniques on the packaging of unhealthy food products. Stronger regulation of these techniques is warranted to protect children’s health, including ongoing monitoring to support potential reform.

## Supplementary material

For supplementary material accompanying this paper visit https://doi.org/10.1017/S136898002300215X
